# Intranasal Administration of Undifferentiated Oligodendrocyte Lineage Cells as a Potential Approach to Deliver Oligodendrocyte Precursor Cells into Brain

**DOI:** 10.3390/ijms221910738

**Published:** 2021-10-04

**Authors:** Ulises Gómez-Pinedo, Jordi A. Matías-Guiu, María Soledad Benito-Martín, Lidia Moreno-Jiménez, Inmaculada Sanclemente-Alamán, Belen Selma-Calvo, Sara Pérez-Suarez, Francisco Sancho-Bielsa, Alejandro Canales-Aguirre, Juan Carlos Mateos-Díaz, Mercedes A. Hernández-Sapiéns, Edwin E. Reza-Zaldívar, Doddy Denise Ojeda-Hernández, Lucía Vidorreta-Ballesteros, Paloma Montero-Escribano, Jorge Matías-Guiu

**Affiliations:** 1 Laboratory of Neurobiology, Institute of Neurosciences, Health Research Institute of the Hospital Clínico San Carlos, Universidad Complutense de Madrid, 28040 Madrid, Spain; msbm65@gmail.com (M.S.B.-M.); lidiamor-92@hotmail.com (L.M.-J.); inmasancle4@gmail.com (I.S.-A.); belselma@ucm.es (B.S.-C.); sarape14@ucm.es (S.P.-S.); matiasguiu@gmail.com (J.M.-G.); 2 Department of Neurology, Institute of Neurosciences IdISSC, Hospital Clinico San Carlos, Universidad Complutense de Madrid, 28040 Madrid, Spain; jordimatiasguiu@hotmail.com (J.A.M.-G.); lvidorreta@alumni.unav.es (L.V.-B.); pmontero84@gmail.com (P.M.-E.); 3 Department of Physiology, Ciudad Real School of Medicine, Universidad de Castilla-La Mancha, 13005 Ciudad Real, Spain; francisco.sancho@uclm.es; 4 Preclinical Evaluation Unit, Medical and Pharmaceutical Biotechnology Unit, CIATEJ-CONACyT, Guadalajara 44270, Mexico; alexcanalex@gmail.com (A.C.-A.); mehernandez_al@ciatej.edu.mx (M.A.H.-S.); edreza_al@ciatej.edu.mx (E.E.R.-Z.); doddydenise@gmail.com (D.D.O.-H.); 5 Department of Industrial Biotechnology, CIATEJ-CONACyT, Zapopan 45019, Mexico; jcmateos@ciatej.mx

**Keywords:** multiple sclerosis, HOG cells, oligodendrocyte precursor cells, oligodendrocytes, intranasal administration, demyelination, remyelination

## Abstract

Oligodendrocyte precursor cell (OPC) migration is a mechanism involved in remyelination; these cells migrate from niches in the adult CNS. However, age and disease reduce the pool of OPCs; as a result, the remyelination capacity of the CNS decreases over time. Several experimental studies have introduced OPCs to the brain via direct injection or intrathecal administration. In this study, we used the nose-to brain pathway to deliver oligodendrocyte lineage cells (human oligodendroglioma (HOG) cells), which behave similarly to OPCs in vitro. To this end, we administered GFP-labelled HOG cells intranasally to experimental animals, which were subsequently euthanised at 30 or 60 days. Our results show that the intranasal route is a viable route to the CNS and that HOG cells administered intranasally migrate preferentially to niches of OPCs (clusters created during embryonic development and adult life). Our study provides evidence, albeit limited, that HOG cells either form clusters or adhere to clusters of OPCs in the brains of experimental animals.

## 1. Introduction

Oligodendrocytes and oligodendrocyte precursor cells (OPC) play a major role in White matter homeostasis [1]. OPCs are generated in the embryonic subventricular zone and migrate to target sites through the central nervous system; in adults, OPCs represent 5% of all brain cells [2,3,4]. This population of immature cells can divide and differentiate into myelinating oligodendrocytes; therefore, they provide progenitor cells for the replacement of oligodendrocytes, which are involved in physiological myelin remodelling [5,6,7,8], adaptive myelination [9,10], and myelin repair following white matter lesions [11], as occurs in multiple sclerosis (MS) and stroke [12]. OPCs react rapidly to myelin damage, mediating in oligodendrocyte regeneration as a compensatory mechanism [13,14]. Following white matter damage, OPCs proliferate and migrate to demyelinated areas, then differentiate into mature oligodendrocytes [15], restoring myelin in the damaged white matter [16]. Remyelination efficiency decreases progressively with age, to the point where remyelination capacity is completely lost [17]; this has significant implications for such chronic diseases as MS and other agerelated disorders, including stroke. The lack of OPCs in older age hinders differentiation into oligodendrocytes [18]; remyelination becomes nearly nonexistent, which makes demyelinated axons susceptible to neurodegeneration, leading to disease progression [19]. Different strategies have been proposed to minimise the impact of age on remyelination by targeting OPCs [20]; one such strategy is transplantation of oligodendrocyte lineage cells, which may act as OPCs when the latter become less abundant [21]. OPC transplantation has been used in patients with spinal cord lesions [22,23,24,25,26], mice with congenital hypomyelination [27], and patients with stroke associated with white matter lesions [28]. It has been suggested that OPCs are able to migrate within the CNS but not from the cerebrospinal fluid (CSF) or the blood, since they are unable to cross the blood-brain barrier; the route of access to demyelinated regions is therefore a relevant consideration in experimental protocols with these cells. Delivery of OPCs through direct injection is most frequently used, although some researchers have recently proposed the intranasal route [29]; cells delivered through this route may reach the CNS [30]. The purpose of this study is to analyse the behaviour of human oligodendroglioma (HOG) cells administered intranasally, with a view to determining whether these cells may be used to restore depleted OPC pools in older individuals and for remyelinating treatment [31].

## 2. Results

### 2.1. In Vitro Study

**Figure 1:**
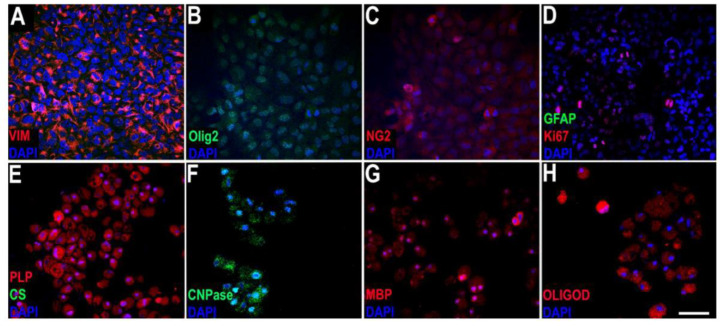
Immunofluorescence photomicrograph showing the expression of oligodendroglial lineage cell markers. (**A**–**G**): immunocytochemical staining for vimentin (**A**), Olig2 (**B**), NG2 (**C**), GFAP + Ki67 (**D**), PLP + glutamine synthetase (**E**), CNPase (**F**), MBP (**G**) and Oligodendrocytes (**H**). Scale bar: 50 µm.

Immunocytochemistry studies of HOG cells revealed positivity for oligodendroglial lineage cell markers, including Olig2, NG2, vimentin, CNPase, PLP, MBP, and Ki67, and negativity for such astrocytic markers as GFAP and glutamine synthetase (Figure 1).

The viability of cell cultures remained at a ratio above 95%. Transfection with plasmid pcDNA3.2/P2A/GFP showed 83% efficiency, a similar value to that reported by the manufacturer of the transfection reagent.

### 2.2. In Vivo Study

None of the animals showed signs of asphyxia, nasal irritation, or haemorrhage after awakening from anaesthesia for intranasal administration of HOG cells. At 30 and 60 days, none of the animals displayed weight loss or any behaviour suggesting alterations in well-being.

### 2.3. Cell Biodistribution

**Figure 2:**
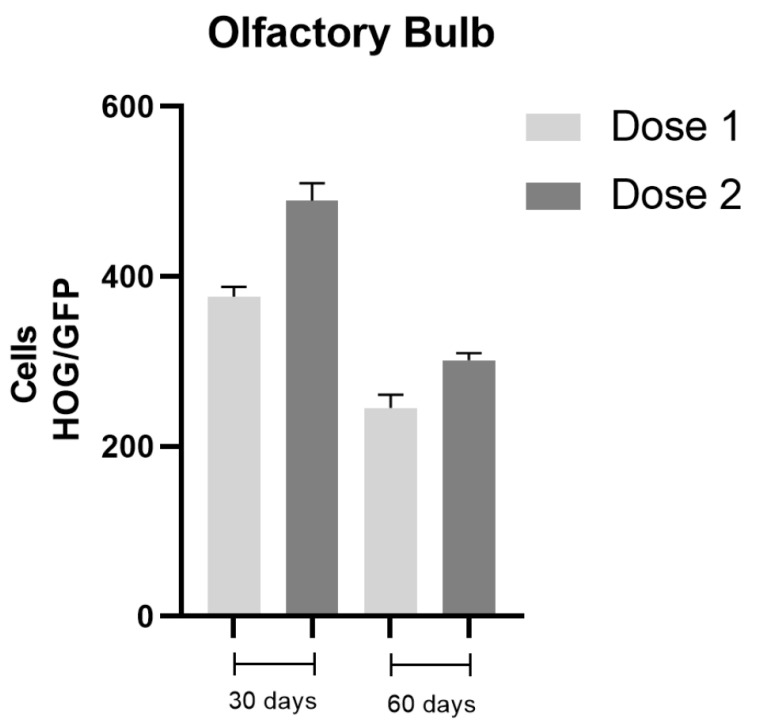
Analysis of cell density in the olfactory bulb. HOG cells were detected in the olfactory bulb, independently of the dose administered; this suggests that the cells are able to cross the cribriform plate, forming clusters near capillaries and apparently integrating into the neuropil. Data are expressed as means ± SE.

Confocal microscopy of the olfactory bulb revealed GFP-labelled cells. HOG cell distribution was similar at both time points, but higher cell density was observed at 30 days after implantation. In the analysis of 10 serial slices at the level of the olfactory bulb, a mean of 376 cells/mm^2^ were detected after administration of dose 1, and 489 cells/mm^2^ in animals receiving dose 2. This suggests that the number of cells crossing the blood-brain barrier from the cribriform plate does not depend on the concentration of cells administered. At 60 days, cell density in the olfactory bulb showed a similar pattern to that observed at 30 days, for both doses, but was lower: 245 cells/mm^2^ for dose 1 and 301 cells/mm^2^ for dose 2 (Figure 2). We were unable to detect HOG cells in the CSF of experimental animals.

At 30 days after implantation, the majority of HOG cells were found in the most superficial layer of the olfactory bulb, which is mainly composed of axons of olfactory receptor neurons and olfactory ensheathing cells. A lower density of GFP-labelled HOG cells was observed in the glomerular layer, with isolated cells in the external plexiform layer. In the olfactory bulb, HOG cells were isolated, not forming clusters.

GFP-labelled cells were also observed in other brain regions, mainly in areas adjacent to the ventricular walls, the base of the corpus callosum, and cortical layer VI, cingulate, striatum/axon bundles, amygdala, areas adjacent to the midbrain, and the walls of the third ventricle; interestingly, more cells were observed in the septum and fimbria of the hippocampus.

**Figure 3:**
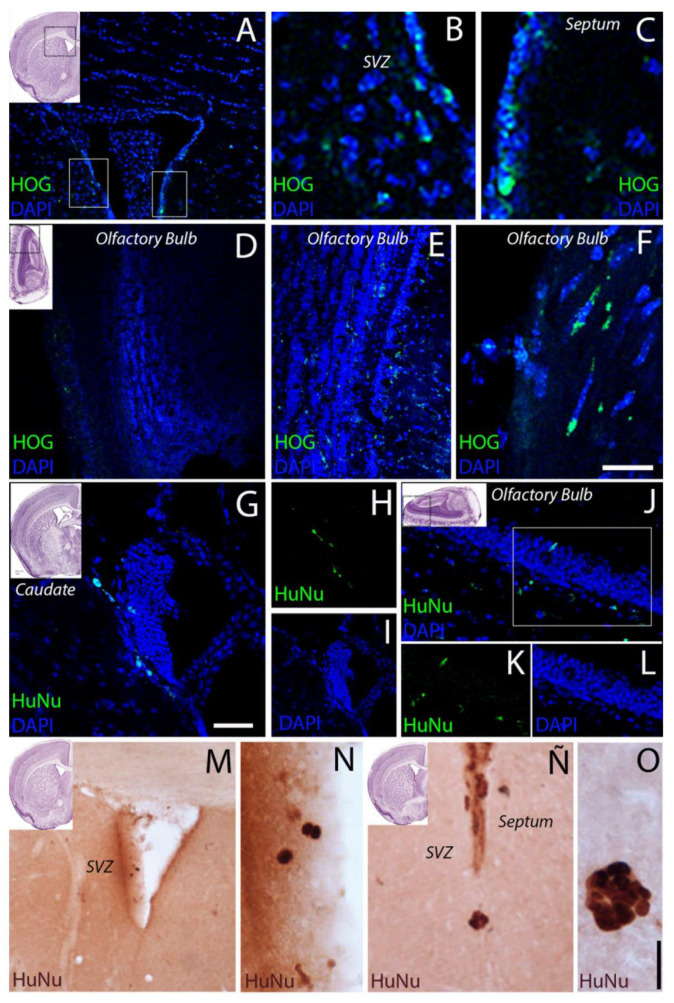
Representative confocal microscopy images of GFP-labelled HOG cells in different brain areas at 60 days after administration of dose 1. (**A**–**C**) In the periventricular region, GFP-labelled HOG cells were observed near the ventricle. (**D**–**F**) In the olfactory bulb, HOG cells were observed in the perigranular cell layer; these presented fusiform (**E**) or spherical morphology (**F**). Scale bar: (**A**) 200 µm; (**B**,**C**) 40 µm; (**D**) 250 µm; (**E**): 40 µm, (**F**): 50 µm. HuNu-positive cells are shown in the caudate region, in the vicinity of the lateral ventricles (**G**–**I**) and in the olfactory bulb (**J**–**L**). In the (**M**–**O**) images, HuNu + cells are shown by IHC-DAB, observing a pattern similar to the markings of the HOG-GFP images in the ventricular region (SVZ-Septum: **M**–**Ñ**) and grouped in small clusters (**O**). Scale bar: HOG-GFP images (**A**) 250 µm; (**B**,**D**) 100 µm; (**E**,**H**) 75 µm; (**C**,**F**) 40 µm. HuNu (**I**,**F**) images: (**G**,**J**) 50 µm; (**H**,**I**,**K**,**L**) 100 µm. (**D**,**A**,**B**) images: (**M**) 200 µm; (**N**) 50 µm; (**Ñ**) 100 µm; (**O**) 25 µm.

**Figure 4:**
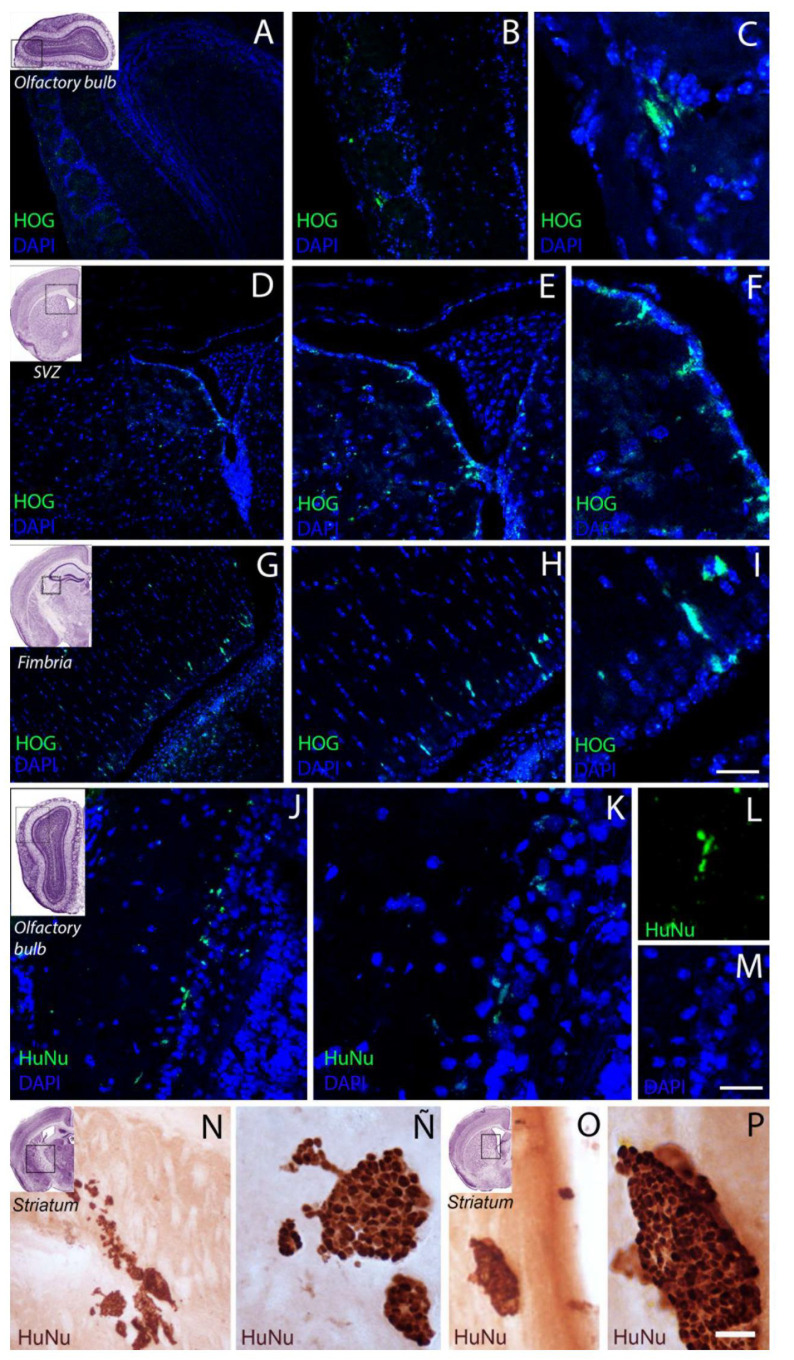
Representative confocal microscopy images of GFP-labelled HOG cells in different brain areas at 60 days after administration of dose 2. (**A**) In the olfactory bulb, HOG cells with small filopodia (**A**–**C**) were observed in the perigranular cell layer. (**D**–**F**) In the periventricular region, GFP-labelled HOG cells were observed near the ventricle; cell density was higher in the septum and (**G**–**I**) In septum, show a fusiform morphology. (**J**–**M**) HuNu-positive cells are shown in the olfactory bulb (image merge **J**,**K**). In (**K**) the labeling is shown in more detail, the (**L**,**M**) images show the individual channels (**L**): HuNu 488 and M nuclei in DAPI. In the (**N**–**P**) images, HuNu + cells are shown by IHC-DAB, observing cells in the ventricular wall and in the striatum region, formed conglomerate cells Scale bar: HOG-GFP images (**A**) 250 µm; (**B**,**D**,**G**) 100 µm; (**E**,**H**) 75 µm; (**C**,**F**) 40 µm; (**G**) 50 µm. HuNu (**I**,**F**) images: (**J**) 50 µm; (**H**,**I**,**K**,**L**) 100 µm; (**M**) 200 µm. (**D**,**A**,**B**) images: (**N**,**O**) 120 µm; (**Ñ**,**P**) 50 µm.

**Figure 5:**
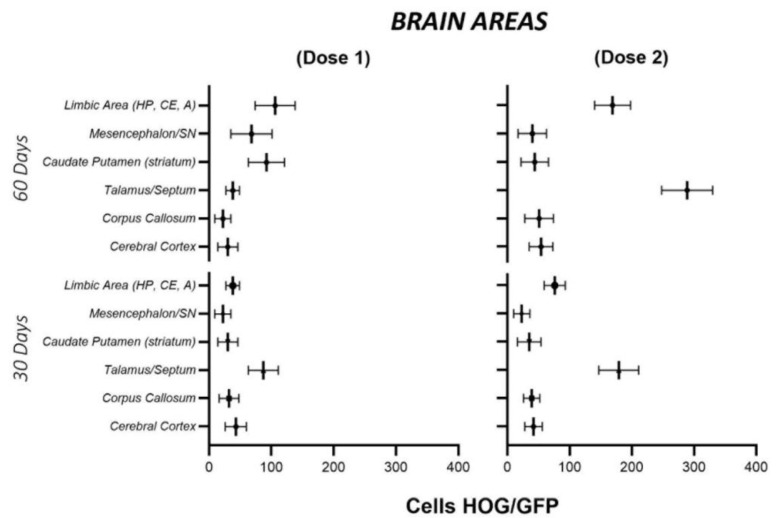
Quantitative analysis of the distribution of GFP-labelled HOG cells in different brain areas. For both doses, higher cell density was observed in the areas adjacent to the hippocampal circuit and the septum. Data are expressed as means ± SE.

At 60 days, a lower density of HOG cells was observed in the olfactory bulb, with few cells in the superficial stratum or glomerular layer. Greater numbers of GFP-labelled cells were observed in other brain regions, particularly in the septum and the fimbria of the hippocampus (Figure 3 and Figure 4). Figure 5 shows the quantitative analysis of the distribution of cells in different brain regions at 30 and 60 days after administration.

**Figure 6:**
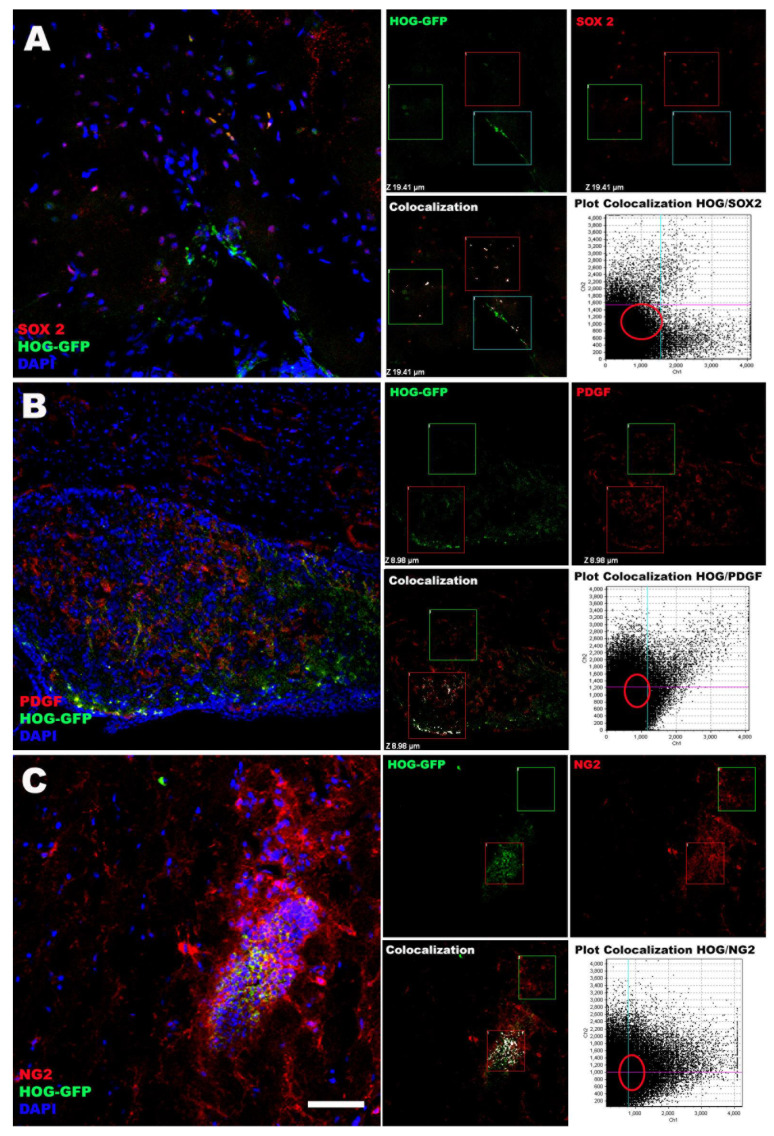
Confocal microscopy images showing immunofluorescence staining with OPC markers (SOX2 (**A**), PDGF (**B**), NG2 (**C**)) in the septum and fimbria. GFP-labelled HOG cells are observed in the proximity of OPCs. Additionally, showing the Pearson colocolalization analysis, where regions of interest are observed that colocalize (threshold, white dotted) and other regions where cells that have positive labeling for OPCs are seen without being positive for the GFP label, these cells being endogenous OPCs (Only SOX2, PDGF or NG2). Scale bar: (**A**,**B**) (50 µm) and (**C**) (25 µm).

Interestingly, at 60 days, cells formed small clusters in such areas as the septum and the fimbria, always in the vicinity of cells with immunohistochemical labelling for SOX2, PDGFR-α or NG2, showing colocolalization of the HOG cells with endogenous OPC markers, in addition, cells that only show the marking for OPCs are observed considering these cells as endogenous OPCs (Figure 6); these are markers of oligodendrocyte progenitor cells, which may remain in a quiescent state in physiological conditions or at basal stages. Haematoxylin-eosin staining did not reveal histopathological signs of hypoxia or inflammation associated with intranasal administration of HOG cells in any animal.

## 3. Discussion

Oligodendrocyte lineage cells capable of differentiating into myelinating oligodendrocytes may be obtained by different means, including differentiation of induced pluripotent stem cells [32,33,34], direct reprogramming of fibroblasts [35,36,37], extraction from the fetal or adult brain, or cells from different tumour-derived cell lines. Among the latter, cell lines from human oligodendroglioma that differentiate into oligodendrocytes with myelinating capacity are most widely used in in vitro studies [38,39,40,41].

In our study, oligodendrocyte lineage cells were administered. In vitro studies have shown that these cells behave like OPCs, expressing markers of precursor cells (vimentin, Ki67) and oligodendrocyte lineage cells (Olig2, NG2, CNPase, MBP, and Ki67). Our in vitro study found that HOG cells behaved like OPCs. HOG cells present similarities with OPCs, and share several antigen markers, including Olig2, NG2, GalC, CNPase, and growth factor receptors [42]. Furthermore, HOG cells may proliferate and differentiate, expressing PLP, MBP, and MOG, and generate myelin [43,44]. These cells have been used in studies on mechanisms related to myelination [45,46,47,48,49] and the influence of the immune response on OPCs [50,51,52], although they present some differences to OPCs, including lack of expression of GFAP or glutamine [53], and the degree of apoptosis [54]. Our in vitro study also confirmed the lack of expression of such astrocytic markers as GFAP or glutamine synthetase.

HOG cells have frequently been used to promote differentiation in vitro and in different culture media [55] in experimental studies aiming to determine their role in neurological disease [56,57]; studies have also been conducted to analyse the impact of genetic variants on OPCs [58,59]. HOG cells have previously been used in multiple sclerosis research [60] and in models of demyelination [61]. Although less evidence is available on the behaviour of HOG cells in vivo, these cells may be helpful in analysing the behaviour of OPCs, which is why they were used in the present study.

### 3.1. Intranasal Administration Enables HOG Cells to Access the CNS

Due to their characteristics, OPCs have classically been administered by direct implantation into the tissue requiring remyelination. In 2004, Windrem et al. [62] implanted fetal and adult human OPCs directly into the corpus callosum of mice, and also to lysolecithin-induced lesions [63]. Webber et al. [64] injected OPCs from rats in to the cortex of rats with periventricular leukomalacia, and Zhou et al. [65] implanted adult human OPCs directly into the corpus callosum and the lateral ventricles of mice, observing that the cells were able to migrate and distribute themselves across the CNS. In a study by Zhong et al. [66], intrathecal administration of human OPCs to neonatal rats with ischaemic white matter lesions promoted remyelination.

We used the intranasal route to implant HOG cells into the brain. This non-invasive route has previously been used to administer drugs to the CNS, since it bypasses the blood-brain barrier [67,68,69]; different intranasal approaches have been proposed to deliver treatments and nanoparticles to the brain [70,71,72,73]. Our study aimed to determine whether HOG cells can reach the CNS through the intranasal route, crossing the cribriform plate, or whether they enter the CNS through the CSF; the latter scenario would be similar to the model described by Zhong et al. [66], who administered OPCs intrathecally. To this end, we transfected cells with plasmid pcDNA3.2-P2A-GFP, enabling visualisation. In our study, HOG cells were detected in the olfactory bulb 30 days after transplantation. The cells were found predominantly in the axons of olfactory receptor neurons and olfactory ensheathing cells, which supports the hypothesis of a route of entry to the CNS through the olfactory pathway rather than the CSF. At 60 days, fewer HOG cells were detected, potentially suggesting cell migration. Some studies have demonstrated the presence of Olig2-expressing OPCs in the olfactory bulb [74,75]; HOG cells may access a structure with resident OPCs.

Therefore, intranasal administration enables HOG cells to access the CNS.

### 3.2. HOG Cells Migrate and Distribute across the CNS

In adults, OPCs are distributed throughout the CNS [76]; however, they are more abundant in certain areas, which serve as sources for cell migration if remyelination is needed. In order to restore OPC pools, we must first determine where cells migrate to and whether they migrate to the sites where OPCs reside. HOG cell density in the olfactory bulb decreased at 60 days, suggesting that the HOG cells detected in the olfactory bulb may have migrated from this site to other areas of the CNS.

GFP-labelled cells were also observed in areas adjacent to the ventricular walls, base of the corpus callosum, cingulate, striatum, amygdala, hippocampus, areas adjacent to the midbrain, and the walls of the third ventricle; this suggests that HOG cells migrated diffusely across the CNS, but showed a preference for areas displaying OPC proliferation during demyelination [77,78,79]. Interestingly, HOG cells migrate both to areas with NG2-expressing OPCs resulting from embryonic cell migration [4] and to niches resulting from differentiation of adult neural stem cells, in the hippocampus and periventricular areas. In some areas, HOG cells relocated close to OPCs, generating cell clusters, which supports the hypothesis that intranasal administration of HOG cells may help restore the pool of OPCs.

### 3.3. Study Limitations

HOG cells have very similar characteristics to endogenous OPCs. However, given their tumoural origin, we should be mindful of the potential risks of administering HOG cells to athymic mice. This similarity enabled us to answer such relevant questions as whether human oligodendroglial lineage cells can reach the CNS through the olfactory epithelium, survive, and distribute themselves across the CNS, and whether intranasal administration is a useful, convenient approach. Our results open the way to new treatment strategies in demyelinating disease, with OPC administration arising as a promising treatment option; the administration of OPCs or induced pluripotent stem cells may promote the repopulation of oligodendroglial niches or the repair of demyelinated axons.

## 4. Material and Methods

### 4.1. Culture of HOG Cells

This cell line is derived from a human oligodendroglioma. HOG cells were cultured in Dulbecco’s Modified Eagle Medium (DMEM [1X], glutamine 2 mM, 10% fetal bovine serum [FBS], and 1% penicillin-streptomycin solution) at 37 °C in a 5% CO_2_ atmosphere and a relative humidity of 95%, in the dark. These are adherent cells with a high proliferation rate, requiring 2–3 passages per week. Passages were carried out under sterile conditions under a laminar flow hood; the protocol is shown in Supplementary Materials Figure S1. The following reagents were used at every passage: phosphate buffered saline (PBS) 1X, Trypsin (TrypLETM express), DMEM, and trypan blue stain (the latter was used for cell counting in a Neubauer chamber). Technical information about the reagents and materials used is provided in Supplementary Materials Tables S1 and S2.

### 4.2. Transfection with Plasmid pcDNA-P2A-GFP

For labelling purposes, cells were transfected with plasmid pcDNA3.2-P2A-GFP. The vector was constructed according to the BioAssays specifications. The transfection protocol was as follows (see Supplementary Materials Figure S2 for a schematic representation): 1.5 × 10^5^ cells were incubated for 24 h in a Petri dish with DMEM (1X) supplemented with 10% FBS and 1% antibiotic-antifungal mixture for adhesion and metabolic activation. After 24 h, the medium was exchanged with serum-free DMEM, and the cells were transfected with a master mix containing serum-free DMEM (1X), TransITR-2020 transfection reagent (Mirus), and plasmid pcDNA3.2-P2A-GFP (the latter 2 at a 4:1 ratio). The master mix was incubated for 30 min at room temperature to allow lipid vesicles to form around the plasmid before cell transfection.

Finally, the cells were incubated at 37 °C and 5% CO_2_ for 48 h, until they were administered intranasally.

### 4.3. Animals

All experimental procedures were carried out at the animal facility of Hospital Clinico San Carlos, located in the Experimental Medicine and Surgery Unit (registration number ES280790000088), Madrid, Spain. The animals were maintained in vivarium conditions (21 ± 1 °C, 12:12 light-dark cycles), with ad libitum access to food and water and environmental enrichment.

We used adult Crl:NU (NCr)-Foxn1nu mice (Charles River Laboratories; Lyon, France), with an average weight of 30 g. Their cages were closed and sterilised, and were changed once per week, in the morning and under laminar flow hood conditions, using sterile sawdust and wood shavings; food and fresh water were also supplied. Crl: NU (NCr) -Foxn1nu mice Immunodeficient animal model are extremely useful in a wide range of biomedical research, including infectious disease, stem cell, immunology and oncology.

Trovan microchips were implanted in all animals to prevent handling errors and for body-weight monitoring. To this end, each individual mouse was first identified using a microchip reader and then weighed on a scale. Both the scale and the reader were cleaned with alcohol prior to use and between animals from different cages to avoid any stressful signals.

### 4.4. Experimental Groups

Our study objective was to test the viability and distribution of HOG cells. Cells were transfected to enable analysis after infusion. Two experimental groups were established: dose 1 (2.2 × 10^4^ HOG cells) and dose 2 (4.4 × 10^4^ HOG cells), with 8 mice per group. In each group, 4 mice were euthanised at 30 days post-implantation and the remaining 4 at 60 days.

### 4.5. Intranasal Administration of HOG Cells

**Figure 7:**
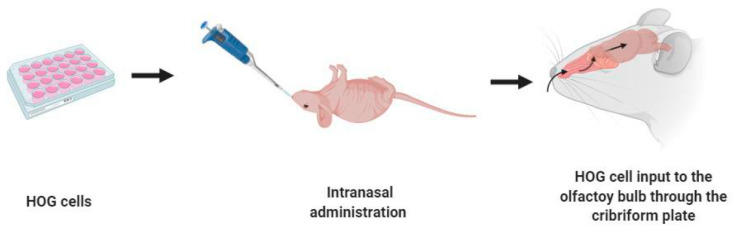
Schematic representation of the HOG cell administration process. HOG cells suspension was prepared and administered to mice intranasally. From the nasal sinus, HOG cells enter the olfactory bulb through the cribriform plate, spreading across the brain. Image created with BioRender.

In the intranasal administration procedure (Figure 7), we first prepared the suspension of transfected HOG cells, and subsequently followed the protocol described by Wei et al. [80]:

The plate containing the transfected cells was treated with trypsin. The enzyme was inactivated with DMEM 1X (10% FBS) and cells centrifuged at 250 g and a room temperature for 5 min. The resulting pellet was resuspended in PBS 0.1 M at physiological pH (7.2–7.4) and cells were counted. The pellet was washed twice with 0.1 M PBS (pH 7.35) to remove remnants of the culture medium; the cell suspension was centrifuged once more and a dilution of 180 μL was prepared with a final concentration for the dose 1 or dose 2.

Mice were administered bovine hyaluronidase 300 IU/mg (H7981, US Biological) prior to administration of HOG cells (oligodendroglial lineage cells). Hyaluronidase was used to increase the permeability of the olfactory epithelium and thus promote passage across the nasal mucosal barrier [80,81,82,83]. A total of 50 μL hyaluronidase solution was prepared, at a final concentration of 100 IU/μL of hyaluronidase in PBS 0.1 M. Mice were anaesthetised with 3% isoflurane, and anaesthesia was maintained with 1.5% isoflurane. Animals were placed in the supine position and their heads were manually held at an angle of 70–90° so that HOG cells would remain in the atrium rather than descending into the pharynx; 1 μL hyaluronidase solution was administered into each nostril. After 15 min, HOG cells were administered at 2 different doses (dose 1 or dose 2), with the animals in the same position: an automatic pipette with a tip for low volumes was used to administer 2 μL of the cell suspension per nostril.

### 4.6. Euthanasia and Perfusion

Animals were euthanised at either 30 or 60 days after injection, with an overdose of intraperitoneal fentanyl and thiopental (0.4 mg/kg and 40–60 mg/kg, respectively). Perfusion was performed with the animals under deep anaesthesia. They were placed in a supine position and the thorax was opened. The diaphragm was cut, and the ribs were gradually removed until reaching the heart. A cannula was inserted into the left ventricle up to the entrance of the aorta, and the right atrium was cut to prevent hypervolaemia. The cannula was used to administer 250 mL saline solution with 250 μL heparin (5000 U) at a flow rate of 15 mL/min, to remove clots (approximately 10 min); 100 mL fixative solution (paraformaldehyde [PFA] 4% [9713.5000, VWR Chemicals], sucrose 7%, PB 0.1 M, and Milli-Q water) was subsequently added for 5–7 min; this procedure was performed with a peristaltic pump (Thermo Fisher Scientific). After perfusion, the brain (starting from occipital to frontal, to avoid damage to the olfactory bulbs), nose, and lungs of each animal were removed and immersed in PFA 4% at room temperature and in agitation for 24 h.

The brains and lungs were fixed in PFA 4% and cryoprotected in sucrose 30% with PBS azide in 3 passages; the last passage included a mixture of sucrose and cryostat resin (OCT) at a 50:50 ratio. The brains were cut sagittally, separating both hemispheres. Cryostat blocks were obtained from each hemisphere, embedded in OCT, and stored at −20 °C until use. Using a cryostat (MICROM HM-505E), the brains were cut into 30-μm longitudinal and coronal sections (right and left hemisphere, respectively). Sections were collected in PBS-azide and processed as free-floating sections. We studied the olfactory bulb, anatomical regions adjacent to the administration site (e.g., nasal cavity), and the brain, to determine the areas of the CNS to which HOG cells had migrated.

### 4.7. Haematoxylin-Eosin Stain

Tissue sections both from the olfactory bulb and from other brain regions were mounted on microscope slides and stained with haematoxylin-eosin, as follows:

Tissue samples were washed twice with Milli-Q water to remove traces of OCT, for 2 min each wash. They were left to stain in haematoxylin for 2 min, after which the remaining stain was rinsed with Milli-Q water for 5 min, using a staining jar. Tissue samples were then stained with eosin for 30 s and rinsed again under running water. Finally, samples were dehydrated with increasing concentrations of ethanol (80 s each), starting with 70% ethanol with 2% acetic acid, and followed by 90% ethanol, 96% ethanol, 100% ethanol, and xylol for 5 min. They were mounted on microscope slides with DPX mounting medium (distyrene, a plasticiser, and xylene).

The stained tissues were studied under a Leitz Laboreux 5 optical microscope, and photomicrographs were taken using a MOTIC 5.0 PX camera and the Motic Images Plus 3.0 computer software. The magnifications used were 6×, 25×, and 40×.

### 4.8. Immunohistochemical and Immunocytochemical Techniques

Immunocytochemistry was performed to confirm the identity of the incubated cells. For this purpose, cells were seeded in treated 8-well chambers (30742079, Eppendorf) (1 × 10^4^ cells/well) and in 24-well Petri dishes (351147, Thermo Fisher Scientific, Madrid, Spain) with untreated covers (4 × 10^4^ cells/well). They were incubated for 72 h, washed in PBS (0.1 M), and fixed in PFA 4%. The cells were kept in PBS-azide (PBS 0.1 M, 0.1% sodium azide, and Milli-Q water) at 4 °C until the immunocytochemistry study, for which they were treated with the following antibodies:

For HOG cells, several immunofluorescence studies were performed using anti- CNPase, anti-Ki67, anti-NG2, anti-MBP, anti-oligodendrocyte, anti-Olig2, and anti-vimentin antibodies. The characteristics of these antibodies are summarised in Supplementary Materials Table S2. Primary antibodies were incubated overnight at 4 °C. The next day, they were washed with PBS-triton-albumin (PTA) and the secondary antibodies were incubated for 2 h at room temperature in the dark. Finally, cells were mounted on FluorSave^TM^ mounting medium (345589-20 mL, Calbiochem, London, UK) to protect the fluorescence.

For tissue samples, immunofluorescence was performed with anti–human nuclei and anti-GFP (DyLight 488) antibodies, on microscope slides. Subsequently, non-specific binding sites were blocked with normal donkey serum (NDS) 3% in PTA (ab7475, Abcam, Cambridge, UK) for 30–90 min at room temperature. Anti–human nuclei and anti-GFP antibodies were incubated overnight at 4 °C and covered with parafilm to prevent them from drying out (anti-GFP antibodies must also be incubated in the dark). Fluorophore was already incorporated; it was washed with PBS (1×); For the identification of endogenous OPCs, the SOX-2, PDGF-a and NG2 antibodies were used. Finally, tissue samples were mounted with the appropriate mounting medium and stored until microscopic observation.

Two different microscopes were used: tissues and cells treated with immunoperoxidase were observed under a Leitz Laboreux 5 optical microscope at 25× and 40× magnification, while tissues and cells treated with immunofluorescence were observed under an Olympus AF-1200 confocal microscope (School of Chemistry, Universidad Complutense of Madrid), at 10×, 20×, and 40× magnification in the case of tissues, and at 60× with an immersion objective in the case of cells.

Fifteen serial sections of mouse brains were collected to analyse the anatomical distribution of GFP-labelled HOG cells in the olfactory bulb and brain. Confocal microscopy was used to study regions of interest in the brain (cerebral cortex, corpus callosum, caudate nucleus, putamen, thalamus/septum, limbic area) for quantitative analysis. In addition, the colocalization analysis was performed using the confocal software, in order to analyze whether the HOG-GFP cells share endogenous OPC’s markers.

## 5. Conclusions

HOG cells, which behave similarly to OPCs in vitro, can reach the CNS when administered intranasally, and are subsequently distributed across the CNS, with a preference for OPC niches of both embryonic and neurogenic origin. Our results suggest that HOG cells either form clusters or adhere to clusters of OPCs.

As we did not use experimental models of disease, the migration of HOG cells through the CNS was not stimulated by inflammatory or demyelinating lesions, but rather was a biological behavior of these cells. This observation may support the administration of HOG cells as a preventive measure, since repopulation of OPC niches may help to prevent age-related OPC depletion.

However, there is a need for future studies addressing two main issues: firstly, whether these cells remain resident and in a quiescent state in OPC niches, their survival times and the factors involved in their survival, and the potential role of OPC-loaded microparticles; and secondly, the capacity of HOG cells to respond to such demyelinating diseases as multiple sclerosis [31] and neuromyelitis optica [84], degenerative diseases such as Alzheimer disease [85], trauma [86], vascular diseases, and primary diseases of myelin, such as Alexander disease [87]. Although the implanted HOG cells can proliferate and differentiate in vitro, questions remain on whether they behave similarly in vivo. In any case, the use of CNS precursor cells constitutes a new therapeutic approach to a number of diseases [88].

## Data Availability

In this section, please provide details regarding where data supporting reported results can be found, including links to publicly archived datasets analyzed or generated during the study. Please refer to suggested Data Availability Statements in section “MDPI Research Data Policies” at https://www.mdpi.com/ethics, accessed on 25 September 2021. You might choose to exclude this statement if the study did not report any data.

## Supplementary Materials

The following are available online at https://www.mdpi.com/article/10.3390/ijms221910738/s1.

## References

[1] Boulanger J.J., Messier C. (2014). From precursors to myelinating oligodendrocytes: Contribution of intrinsic and extrinsic factors to white matter plasticity in the adult brain. Neuroscience.

[2] Tsai H.H., Niu J., Munji R., Davalos D., Chang J., Zhang H., Tien A.C., Kuo C.J., Chan J.R., Daneman R. (2016). Oligodendrocyte precursors migrate along vasculature in the developing nervous system. Science.

[3] Levine J.M., Reynolds R., Fawcett J.W. (2001). The oligodendrocyte precursor cell in health and disease. Trends Neurosci..

[4] Dawson M.R., Polito A., Levine J.M., Reynolds R. (2003). NG2-expressing glial progenitor cells: An abundant and widespread population of cycling cells in the adult rat CNS. Mol. Cell. Neurosci..

[5] Young K.M., Psachoulia K., Tripathi R.B., Dunn S.J., Cossell L., Attwell D., Tohyama K., Richardson W.D. (2013). Oligodendrocyte dynamics in the healthy adult CNS: Evidence for myelin remodeling. Neuron.

[6] Hill R.A., Li A.M., Grutzendler J. (2018). Lifelong cortical myelin plasticity and age-related degeneration in the live mammalian brain. Nat. Neurosci..

[7] Hughes E.G., Orthmann-Murphy J.L., Langseth A.J., Bergles D.E. (2018). Myelin remodeling through experience-dependent oligodendrogenesis in the adult somatosensory cortex. Nat. Neurosci..

[8] Tripathi R.B., Jackiewicz M., McKenzie I.A., Kougioumtzidou E., Grist M., Richardson W.D. (2017). Remarkable Stability of Myelinating Oligodendrocytes in Mice. Cell Rep..

[9] Gibson E.M., Purger D., Mount C.W., Goldstein A.K., Lin G.L., Wood L.S., Inema I., Miller S.E., Bieri G., Zuchero J.B. (2014). Neuronal activity promotes oligodendrogenesis and adaptive myelination in the mammalian brain. Science.

[10] Mitew S., Gobius I., Fenlon L.R., McDougall S.J., Hawkes D., Xing Y.L., Bujalka H., Gundlach A.L., Richards L.J., Kilpatrick T.J. (2018). Pharmacogenetic stimulation of neuronal activity increases myelination in an axonspecific manner. Nat. Commun..

[11] Zawadzka M., Rivers L.E., Fancy S.P.J., Zhao C., Tripathi R., Jamen F., Young K., Goncharevich A., Pohl H., Rizzi M. (2010). CNS resident glial progenitor/stem cells produce Schwann cells as well as oligodendrocytes during repair of CNS demyelination. Cell Stem Cell.

[12] Matias-Guiu J., Martinez-Vazquez J., Ruibal A., Colomer R., Boada M., Codina A. (1986). Myelin basic protein and creatine kinase BB isoenzyme as CSF markers of intracranial tumors and stroke. Acta Neurol. Scand..

[13] Maki T., Liang A.C., Miyamoto N., Lo E.H., Arai K. (2013). Mechanisms of oligodendrocyte regeneration from ventricular-subventricular zone-derived progenitor cells in White matter diseases. Front. Cell. Neurosci..

[14] Zhang R., Chopp M., Zhang Z.G. (2013). Oligodendrogenesis after cerebral ischemia. Front. Cell. Neurosci..

[15] Hughes E.G., Kang S.H., Fukaya M., Bergles D.E. (2013). Oligodendrocyte progenitors balance growth with self-repulsion to achieve homeostasis in the adult brain. Nat. Neurosci..

[16] Itoh K., Maki T., Lok J., Arai K. (2015). Mechanisms of cell-cell interaction in oligodendrogenesis and remyelination after stroke. Brain Res..

[17] Sim F.J., Zhao C., Penderis J., Franklin R.J.M. (2002). The age-related decrease in CNS remyelination efficiency is attributable to an impairment of both oligodendrocyte progenitor recruitment and differentiation. J. Neurosci..

[18] Shen S., Sandoval J., Swiss V.A., Li J., Dupree J., Franklin R.J.M., Casaccia-Bonnefil P. (2008). Age-dependent epigenetic control of differentiation inhibitors is critical for remyelination efficiency. Nat. Neurosci..

[19] Franklin R.J.M., Edgar J.M., Smith K.J., Chrles ffrench-Constant (2012). Neuroprotection and repair in multiple sclerosis. Nat. Rev. Neurol..

[20] Neumann B., Baror R., Zhao C., Segel M., Dietmann S., Rawji K.S., Foerster S., McClain C.R., Chalut K., van Wijngaarden P. (2019). Metformin Restores CNS Remyelination Capacity by Rejuvenating Aged Stem Cells. Cell Stem Cell.

[21] Egawa N., Takase H., Josephine L., Takahashi R., Arai K. (2016). Clinical application of oligodendrocyte precursor cells for cell-based therapy. Brain Circ..

[22] Manley N.C., Priest C.A., Denham J., Wirth E.D., Lebkowski J.S. (2017). Human Embryonic Stem Cell-Derived Oligodendrocyte Progenitor Cells: Preclinical Efficacy and Safety in Cervical Spinal Cord Injury. Stem Cells Trans. Med..

[23] Priest C.A., Manley N.C., Denham J., Wirth E.D., Lebkowski J.S. (2015). Preclinical safety of human embryonic stem cell-derived oligodendrocyte progenitors supporting clinical trials in spinal cord injury. Regen. Med..

[24] All A.H., Gharibani P., Gupta S., Bazley F.A., Pashai N., Chou B.K., Shah S., Resar L.M., Cheng L., Gearhart J.D. (2015). Early intervention for spinal cord injury with human induced pluripotent stem cells oligodendrocyte progenitors. PLoS ONE.

[25] Kawabata S., Takano M., Numasawa-Kuroiwa Y., Itakura G., Kobayashi Y., Nishiyama Y., Sugai K., Nishimura S., Iwai H., Isoda M. (2016). Grafted Human iPS Cell-Derived Oligodendrocyte Precursor Cells Contribute to Robust Remyelination of Demyelinated Axons after Spinal Cord Injury. Stem Cell Rep..

[26] Karimi-Abdolrezaee S., Eftekharpour E., Wang J., Morshead C.M., Fehlings M.G. (2006). Delayed transplantation of adult neural precursor cells promotes remyelination and functional neurological recovery after spinal cord injury. J. Neurosci..

[27] Wang J., Chao F., Han F., Zhang G., Xi Q., Li J., Jiang H., Wang J., Yu G., Tian M. (2013). Pet demonstrates functional recovery after transplantation of induced pluripotent stem cells in a rat model of cerebral ischemic injury. J. Nucl. Med..

[28] Yu X., Wu H., Zhao Y., Guo Y., Chen Y., Dong P., Mu Q., Wang X., Wang X. (2018). Bone marrow mesenchymal stromal cells alleviate brain white matter injury via the enhanced proliferation of oligodendrocyte progenitor cells in focal cerebral ischemic rats. Brain Res..

[29] Matias-Guiu J., Matias-Guiu J.A., Montero-Escribano P., Barcia J.A., Canales- Aguirre A.A., Mateos-Diaz J.C., Gomez-Pinedo U. (2020). Particles Containing Cells as a Strategy to Promote Remyelination in Patients With Multiple Sclerosis. Front. Neurol..

[30] Uchegbu I., Wang Z., Xiong G., Tsang A., Schatzlein A. (2019). Nose to brain delivery. J. Pharmacol. Exp. Ther..

[31] Matias-Guiu J., Gomez-Pinedo U., Matias-Guiu J.A. (2017). News in multiple sclerosis: Remyelination as a therapeutic target. Med. Clin..

[32] Czepiel M., Balasubramaniyan V., Schaafsma W., Stancic M., Mikkers H., Huisman C., Boddeke E., Copray S. (2011). Differentiation of induced pluripotent stem cells into functional oligodendrocytes. Glia.

[33] Wang S., Bates J., Li X., Schanz S., Chandler-Militello D., Levine C., Maherali N., Studer L., Hochedlinger K., Windrem M. (2013). Human iPSC-derived oligodendrocyte progenitor cells can myelinate and rescue a mouse model of congenital hypomyelination. Cell Stem Cell.

[34] Sim F.J., McClain C.R., Schanz S.J., Protack T.L., Windrem M.S., Goldman S.A. (2011). CD140a identifies a population of highly myelinogenic, migration-competent and efficiently engrafting human oligodendrocyte progenitor cells. Nat. Biotechnol..

[35] Nazm Bojnordi M., Ghasemi H.H., Akbari E. (2014). Remyelination after lysophosphatidyl choline-induced demyelination is stimulated by bone marrow stromal cell-derived oligoprogenitor cell transplantation. Cells Tissues Organs.

[36] Yang N., Zuchero J.B., Ahlenius H., Marro S., Ng Y.H., Vierbuchen T., Hawkins J.S., Geissler R., Barres B.A., Wernig M. (2013). Generation of oligodendroglial cells by direct lineage conversion. Nat. Biotechnol..

[37] Kim J.B., Lee H., Arauzo-Bravo M.J., Hwang K., Nam D., Park M.R., Zaehres H., Park K.I., Lee S.J. (2015). Oct4- induced oligodendrocyte progenitor cells enhance functional recovery in spinal cord injury model. EMBO J..

[38] Post G.R., Dawson G. (1992). Characterization of a cell line derived from a human oligodendroglioma. Mol. Chem. Neuropathol..

[39] Dawson G., Dawson S.A., Post G.R. (1993). Regulation of phospholipase D activity in a human oligodendroglioma cell line (HOG). J. Neurosci. Res..

[40] Buntinx M., Vanderlocht J., Hellings N., Vandenabeele F., Lambrichts I., Raus J., Ameloot M., Stinissen P., Steels P. (2003). Characterization of three human oligodendroglial cell lines as a model to study oligodendrocyte injury: Morphology and oligodendrocyte-specific gene expression. J. Neurocytol..

[41] de Arriba Zerpa G.A., Saleh M.C., Fernandez P.M., Guillou F., Espinosa de los Monteros A., de Vellis J., Zakin M.M., Baron B. (2000). Alternative splicing prevents transferrin secretion during differentiation of a human oligodendrocyte cell line. J. Neurosci. Res..

[42] Persson A.I., Petritsch C., Swartling F.J., Itsara M., Sim F.J., Auvergne R., Goldenberg D.D., Vandenberg S.R., Nguyen K.N., Yakovenko S. (2010). Non-stem cell origin for oligodendroglioma. Cancer Cell.

[43] Dubois-Dalcq M., Behar T., Hudson L., Lazzarini R.A. (1986). Emergence of three myelin proteins in oligodendrocytes cultured without neurons. J. Cell Biol..

[44] Bello-Morales R., Crespillo A.J., Garcia B., Dorado L.A., Martin B., Tabares E. (2014). The effect of cellular differentiation on HSV-1 infection of oligodendrocytic cells. PLoS ONE.

[45] Post G.R., Dawson G. (1992). Regulation of carbachol-and histamine-induced inositol phospholipid hydrolysis in a human oligodendroglioma. Glia.

[46] Chen H.L., Chew L.J., Packer R.J., Gallo V. (2013). Modulation of the Wnt/beta-catenin pathway in human oligodendroglioma cells by Sox17 regulates proliferation and differentiation. Cancer Lett..

[47] Qin J., Goswami R., Dawson S., Dawson G. (2008). Expression of the receptor for advanced glycation end products in oligodendrocytes in response to oxidative stress. J. Neurosci. Res..

[48] Testai F.D., Landek M.A., Goswami R., Ahmed M., Dawson G. (2004). Acid sphingomyelinase and inhibition by phosphate ion: Role of inhibition by phosphatidylmyo- inositol 3,4,5-triphosphate in oligodendrocyte cell signaling. J. Neurochem..

[49] Testai F.D., Landek M.A., Dawson G. (2004). Regulation of sphingomyelinases in cells of the oligodendrocyte lineage. J. Neurosci. Res..

[50] Buntinx M., Moreels M., Vandenabeele F., Lambrichts I., Raus J., Steels P., Stinissen P., Ameloot M. (2004). Cytokine-induced cell death in human oligodendroglial cell lines: I. Synergistic effects of IFN-gamma and TNF-alpha on apoptosis. J. Neurosci. Res..

[51] Buntinx M., Gielen E., Van Hummelen P., Raus J., Ameloot M., Steels P., Stinissen P. (2004). Cytokine-induced cell death in human oligodendroglial cell lines. II: Alterations in gene expression induced by interferon-gamma and tumor necrosis factor-alpha. J. Neurosci. Res..

[52] Starost L., Lindner M., Herold M., Xu Y.K.T., Drexler H.C.A., Hes K., Ehrlich M., Ottoboni L., Ruffini F., Stehling M. (2020). Extrinsic immune cell-derived, but not intrinsic oligodendroglial factors contribute to oligodendroglial differentiation block in multiple sclerosis. Acta Neuropathol..

[53] Chiu M., Taurino G., Bianchi M.G., Ottaviani L., Andreoli R., Ciociola T., Lagrasta C.A., Tardito S., Bussolati O. (2018). Oligodendroglioma Cells Lack Glutamine Synthetase and Are Auxotrophic for Glutamine, but Do not Depend on Glutamine Anaplerosis for Growth. Int. J. Mol. Sci..

[54] Das P., Estephan R., Banerjee P. (2003). Apoptosis is associated with an inhibition of aminophospholipid translocase (APTL) in CNS-derived HN2-5 and HOG cells and phosphatidylserine is a recognition molecule in microglial uptake of the apoptotic HN2-5 cells. Life Sci..

[55] De Keijn K.M.A., Zuure W.A., Peijnenborg J., Hevelmans J.M., Martens G.J.M. (2019). Reappraisal of Human HOG and MO3.13 cell lines as model to study oligodendrocyte functioning. Cells.

[56] Lopez-Guerrero J.A., de la Nuez C., Praena B., Sanchez-Leon E., Krummenacher C., Bello-Morales R. (2020). Herpes Simplex Virus 1 Spread in Oligodendrocytic Cells Is Highly Dependent on MAL Proteolipid. J. Virol..

[57] Podbielska M., Szulc Z.M., Kurowska E., Hogan E.L., Bielawski J., Bielawska A., Bhat N.R. (2016). Cytokine-induced release of ceramide-enriched exosomes as a mediator of cell death signaling in an oligodendroglioma cell line. J. Lipid Res..

[58] Erdem-Eraslan L., Heijsman D., de Wit M., Kremer A., Sacchetti A., van der Spek P.J., Smitt P.A.S., French P.J. (2015). Tumor-specific mutations in low-frequency genes affect their functional properties. J. Neurooncol..

[59] Jenkins R.B., Blair H., Ballman K.V., Giannini C., Arusell R.M., Law M., Flynn H., Passe S., Felten S., Brown P.D. (2006). A t(1;19)(q10;p10) mediates the combined deletions of 1p and 19q and predicts a better prognosis of patients with oligodendroglioma. Cancer Res..

[60] Dasgupta S., Ray S.K. (2017). Diverse biological functions of sphingolipids in the CNS: Ceramide and Sphingosine regulate myelination in developing brain but stimulate demyelination during pathogenesis of multiple sclerosis. J. Neurol. Psychol..

[61] Martinez-Pinilla E., Rubio-Sardon N., Villar-Conde S., Navarro G., Del Valle E., Tolivia J., Franco R., Navarro A. (2021). Cuprizone-Induced Neurotoxicity in Human Neural Cell Lines Is Mediated by a Reversible Mitochondrial Dysfunction: Relevance for Demyelination Models. Brain Sci..

[62] Windrem M.S., Nunes M.C., Rashbaum W.K., Schwartz T.H., Goodman R.A., McKhann G., Roy N.S., Goldman S.A. (2004). Fetal and adult human oligodendrocyte progenitor cell isolates myelinate the congenitally dysmyelinated brain. Nat. Med..

[63] Windrem M.S., Roy N.S., Wang J., Nunes M., Benraiss A., Goodman R., McKhann G.M., Goldman S.A. (2002). Progenitor cells derived from the adult human subcortical white matter disperse and differentiate as oligodendrocytes within demyelinated lesions of the rat brain. J. Neurosci. Res..

[64] Webber D.J., van Blitterswijk M., Chandran S. (2009). Neuroprotective effect of oligodendrocyte precursor cell transplantation in a long-term model of periventricular leukomalacia. Am. J. Pathol..

[65] Zhou H., Lu S., Li K., Yang Y., Hu C., Wang Z., Ye D., Guan Q. (2021). Study on the Safety of Human Oligodendrocyte Precursor Cell Transplantation in Young Animals and Its Efficacy on Myelination. Stem Cells Dev..

[66] Zhong X., Luan Z., Zang J., Guan Q., Yang Y.X., Wang Q., Shi Y. (2021). Protective effect of transplantation of human oligodendrocyte precursor cells in a rat model of White matter injury. Zhongguo Dang Dai Er Ke Za Zhi.

[67] Espinoza L.C., Vacacela M., Clares B., Garcia M.L., Fabrega M.J., Calpena A.C. (2018). Development of a Nasal Donepezil-loaded Microemulsion for the Treatment of Alzheimer’s Disease: In vitro and ex vivo Characterization. CNS Neurol. Disord. Drug Targets.

[68] Dhuria S.V., Hanson L.R., Frey W.H. (2010). Intranasal delivery to the central nervous system: Mechanisms and experimental considerations. J. Pharm. Sci..

[69] Pardeshi C.V., Belgamwar V.S. (2013). Direct nose to brain drug delivery via integrated nerve pathways bypassing the blood-brain barrier: An excellent platform for brain targeting. Expert Opin. Drug Deliv..

[70] Rassu G., Porcu E.P., Fancello S., Obinu A., Senes N., Galleri G., Migheli R., Gavini E., Giunchedi P. (2019). Intranasal Delivery of Genistein-Loaded Nanoparticles as a Potential Preventive System against Neurodegenerative Disorders. Pharmaceutics.

[71] Rassu G., Soddu E., Posadino A.M., Pintus G., Sarmento B., Giunchedi P., Gavini E. (2017). Nose-to-brain delivery of BACE1 siRNA loaded in solid lipid nanoparticles for Alzheimer’s therapy. Colloids Surf. B Biointerfaces.

[72] Sonvico F., Clementino A., Buttini F., Colombo G., Pescina S., Staniscuaski Guterres S., Raffin Pohlmann A., Nicoli S. (2018). Surface-Modified Nanocarriers for Nose-to-Brain Delivery: From Bioadhesion to Targeting. Pharmaceutics.

[73] Rassu G., Gavini E., Carta A., Obinu A., Porcu E.P., Giunchedi P. (2018). Hydroxypropyl-β-Cyclodextrin Formulated in Nasal Chitosan Microspheres as Candidate Therapeutic Agent in Alzheimer’s Disease. Curr. Drug Deliv..

[74] Wang H., Xu L., Lai C., Hou K., Chen J., Guo Y., Sambangi A., Swaminathan S., Xie C., Wu Z. (2021). Region-specific distribution of Olig2-expressing astrocytes in adult mouse brain and spinal cord. Mol. Brain.

[75] Marei H.E., Shouman Z., Althani A., Afifi N., A A.E., Lashen S., Hasan A., Caceci T., Rizzi R., Cenciarelli C. (2018). Differentiation of human olfactory bulb-derived neural stem cells toward oligodendrocyte. J. Cell. Physiol..

[76] Kuhn S., Gritti L., Crooks D., Dombrowski Y. (2019). Oligodendrocytes in Development, Myelin Generation and Beyond. Cells.

[77] Garcia-Martinez Y., Sanchez-Huerta K.B., Pacheco-Rosado J. (2020). Quantitative characterization of proliferative cells subpopulations in the hilus of the hippocampus of adult Wistar rats: An integrative study. J. Mol. Histol..

[78] Liu Q., Lv H.W., Yang S., He Y.Q., Ma Q.R., Liu J. (2020). NEP1-40 alleviates behavioral phenotypes and promote oligodendrocyte progenitor cell differentiation in the hippocampus of cuprizone-induced demyelination mouse model. Neurosci. Lett..

[79] Baxi E.G., DeBruin J., Jin J., Strasburger H.J., Smith M.D., Orthmann-Murphy J.L., Schott J.T., Fairchild A.N., Bergles D.E., Calabresi P.A. (2017). Lineage t acing reveals dynamic changes in oligodendrocyte precursor cells following cuprizone-induced demyelination. Glia.

[80] Wei N., Yu S.P., Gu X., Taylor T.M., Song D., Liu X.F., Wei L. (2013). Delayed intranasal delivery of hypoxic-preconditioned bone marrow mesenchymal stem cells enhanced cell homing and therapeutic benefits after ischemic stroke in mice. Cell Transplant..

[81] Danielyan L., Schafer R., von Ameln-Mayerhofer A., Buadze M., Geisler J., Klopfer T., Burkhardt U. (2009). Intranasal delivery of cells to the brain. Eur. J. Cell Biol..

[82] Beigi Boroujeni F., Pasbakhsh P., Mortezaee K., Pirhajati V., Alizadeh R., Aryanpour R., Madadi S., Ragerdi Kashani I. (2020). Intranasal delivery of SDF-1α- preconditioned bone marrow mesenchymal cells improves remyelination in the cuprizone-induced mouse model of multiple sclerosis. Cell Biol. Int..

[83] Polito A., Reynolds R. (2005). NG2-expressing cells as oligodendrocyte progenitors in thenormal and de myelinated adult central nervous system. J. Anat..

[84] Gomez-Pinedo U., Garcia-Avila Y., Gallego-Villarejo L., Matias-Guiu J.A., Benito- Martin M.S., Esteban-Garcia N., Sanclemente-Alamán I., Pytel V., Moreno-Jiménez L., Sancho-Bielsa F. (2021). Sera from Patients with NMOSD Reduce the Differentiation Capacity of Precursor Cells in the Central Nervous System. Int. J. Mol. Sci..

[85] Chacon-De-La-Rocha I., Fryatt G., Rivera A.D., Verkhratsky A., Raineteau O., Gomez-Nicola D., Butt A.M. (2020). Accelerated Dystrophy and Decay of Oligodendrocyte Precursor Cells in the APP/PS1 Model of Alzheimer’s-Like Pathology. Front. Cell. Neurosci..

[86] Chancellor K.B., Chancellor S.E., Duke-Cohan J.E., Huber B.R., Stein T.D., Alvarez V.E., Okaty B.W., Dymecki S.M., McKee A.C. (2021). Altered oligodendroglia and astroglia in chronic traumatic encephalopathy. Acta Neuropathol..

[87] Gomez-Pinedo U., Sirerol-Piquer M.S., Duran-Moreno M., Garcia-Verdugo J.M., Matias-Guiu J. (2017). Alexander Disease Mutations Produce Cells with Coexpression of Glial Fibrillary Acidic Protein and NG2 in Neurosphere Cultures and Inhibit Differentiation into Mature Oligodendrocytes. Front. Neurol..

[88] Ramos-Zuniga R., Guerrero-Cazares H., Gomez-Pinedo U., Matias-Guiu J. (2021). The Use of Biomaterials With Stem and Precursor Cells in Diseases of the Central Nervous System; A Step to Clinical Trials. Front. Neurol..

