# Supplementary material for: Intranasal Administration of Undifferentiated Oligodendrocyte Lineage Cells as a Potential Approach to Deliver Oligodendrocyte Precursor Cells into Brain
Source: Int J Mol Sci. 2021 Oct 4;22(19):10738. doi: 10.3390/ijms221910738 (PMC8509516; doi:10.3390/ijms221910738)
Supplement: Supplementary file 1 [file ijms-22-10738-s001.zip › ijms-1394224-supplementary.pdf]

Supplementary Figure S1. Schematic representation of our cell passaging protocol. Created with BioRender.

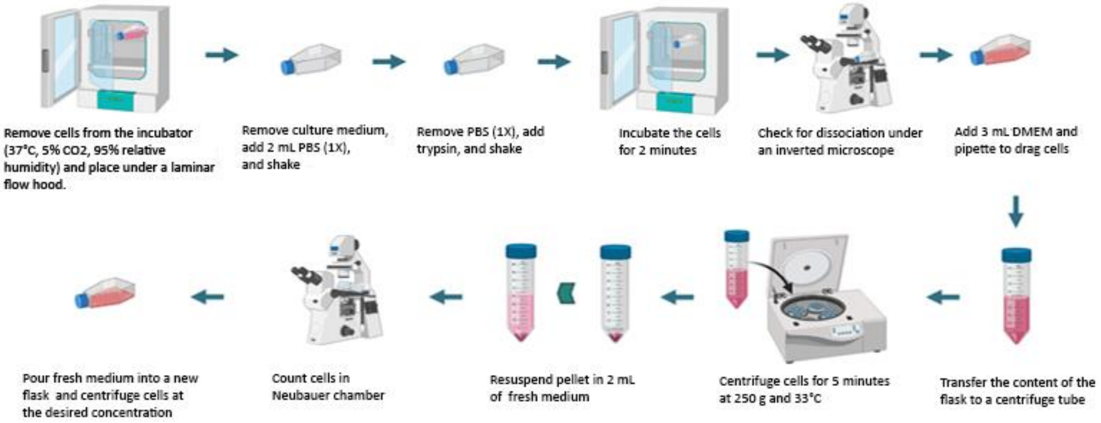

Supplementary Figure S2. (A) Schematic representation of the construction of expression vector pcDNA3.2-P2A-GFP with Myc epitope tag peptide; courtesy of BioAssays. (B) Cell transfection protocol used to introduce plasmid pcDNA3.2-P2A-GFP into HOG cells; created with BioRender

2A

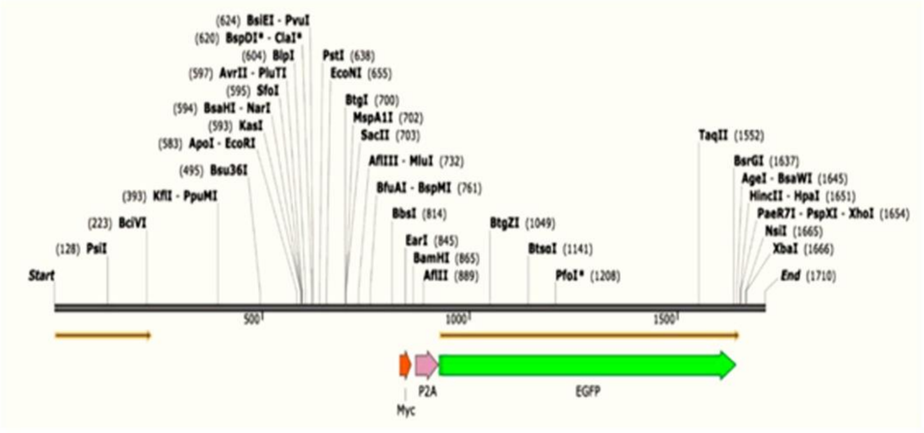

2B

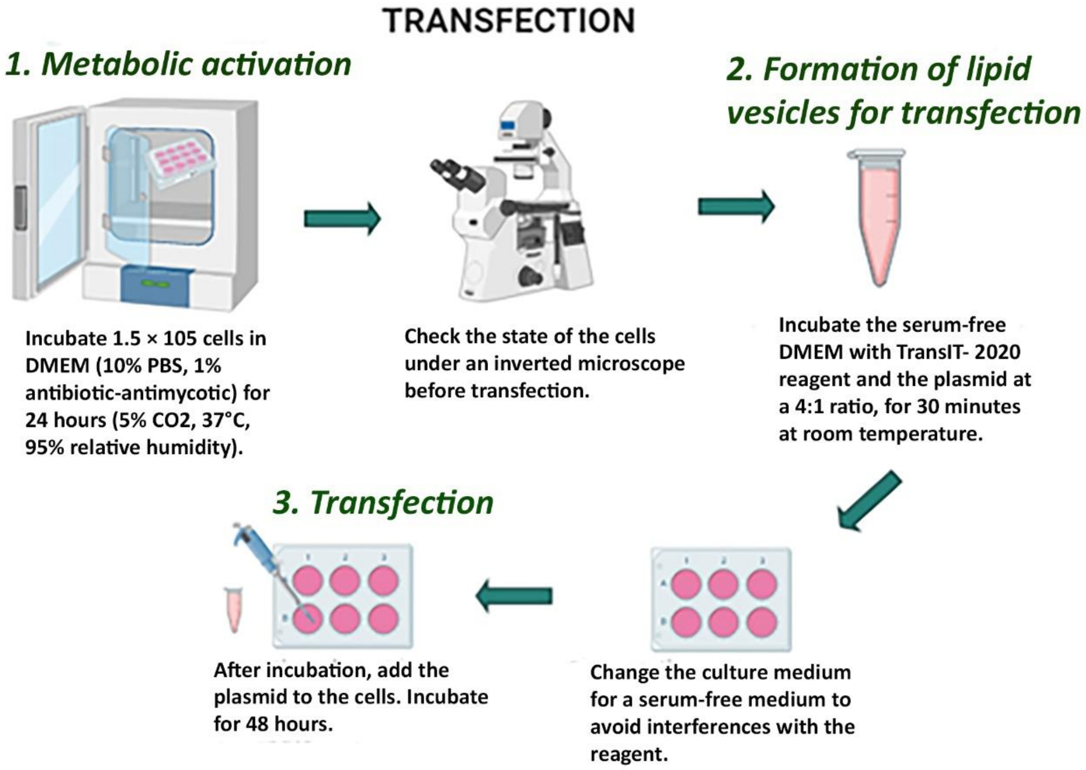

Supplementary Table S1. Technical data on the reagents and materials used

Reagent/material	***Manufacturer***	***Reference***
PBS	GIBCO	10270-106
TrypE™express	GIBCO	12604-013
DMEM	GIBCO	11320-074
FBS	GIBCO	10270-106
Cell culture flask T-25	Eppendorf	0030 710.126
Falcon 15 mL centrifuge tubes	Thermo Fisher Scientific	339650
10 mL pipettes	Fisherbrand	13-676-10J

Supplementary Table S2. Characteristics of the primary and secondary antibodies and fluorophores used in immunohistochemistry

Name	Dilution	Manufacturer	Reference	Observations
**Anti-CNPase**	1:400	Abcam	AB44726	2',3'-cyclic-nucleotide 3'-phosphodiesterase marker; present in myelinating cells.
**Anti-Ki67**	1:500	Abcam	AB16667	Proliferation marker
**Anti-MBP**	1:1000	Merck Millipore	AB5864	Myelin marker
**Anti-oligodendrocytes**	1:500	Merck Millipore	MAB1580	Oligodendrocyte and oligodendrocyte myelin marker
**Anti-NG2**	1:500	Abcam	AB129051	Immature oligodendrocyte marker
**Anti-Olig2**	1:500	Merck Millipore	AB9610	Marker of oligodendrocytes in early stages of maturation
**Anti-vimentin**	1:200	Abcam	AB24525	Marker of intermediate filaments in progenitor cells
**Anti-PDGFRA**	1:200	Boster	PB9771	Platelet-derived growth factor receptor alpha; progenitor oligodendrocyte marker
**Anti-glutamine synthetase**	1:400	Abcam	AB73593	Astrocyte marker
**Anti-GFAP**	1:800	Abcam	AB68428	Astrocyte marker
**Anti–c-Myc tag**	1:250	Rockland Immunochemicals	600-401-381	Myc epitope marker
**Anti–human nuclei**	1:200	Merck Millipore	MAB1281	Human cell nuclei marker
**Anti-GFP DyLight 488**	1:5000	Rockland Immunochemicals	200-341-215	Green fluorescent protein marker, conjugated with secondary antibodies (488 nm)
**Goat anti-mouse IgG 488**	1:500	Thermo Fisher Scientific	A11029	Used against anti-CNPase antibodies
**Goat anti-mouse IgG 555**	1:500	Thermo Fisher Scientific	A21424	Used against anti-oligodendrocyte antibodies
**Goat anti-rabbit IgG 488**	1:500	Thermo Fisher Scientific	A11034	Used against anti-MBP, anti-NG2, and anti-Olig2 antibodies
**Goat anti-rabbit IgG 555**	1:500	Thermo Fisher Scientific	A21428	Used against anti-Ki67 antibodies
**Goat anti-chicken IgY 555**	1:500	Thermo Fisher Scientific	A21437	Used against anti-vimentin antibodies
**DAPI**	1:3000	BioLegend	422801	Nuclear marker visible at 405 nm
**DRAQ**	1:3000	Abcam	AB108410	Nuclear marker visible at 647 nm
